# Structural adjustment programmes and infectious disease mortality

**DOI:** 10.1371/journal.pone.0270344

**Published:** 2022-07-15

**Authors:** Elias Nosrati, Jennifer B. Dowd, Michael Marmot, Lawrence P. King

**Affiliations:** 1 Fafo Institute for Labour and Social Research, Oslo, Norway; 2 Leverhulme Centre for Demographic Science, University of Oxford, Oxford, United Kingdom; 3 Institute of Health Equity, University College London, London, United Kingdom; 4 Department of Economics, University of Massachusetts, Amherst, MA, United States of America; University of Georgia, UNITED STATES

## Abstract

International financial organisations like the International Monetary Fund (IMF) play a central role in shaping the developmental trajectories of fiscally distressed countries through their conditional lending schemes, known as ‘structural adjustment programmes’. These programmes entail wide-ranging domestic policy reforms that influence local health and welfare systems. Using novel panel data from 187 countries between 1990 and 2017 and an instrumental variable technique, we find that IMF programmes lead to over 70 excess deaths from respiratory diseases and tuberculosis per 100,000 population and that IMF-mandated privatisation reforms lead to over 90 excess deaths per 100,000 population. Thus structural adjustment programmes, as currently designed and implemented, are harmful to population health and increase global infectious disease burdens.

## Introduction

In the wake of the COVID-19 pandemic, much scientific effort has been devoted to better understanding the institutional determinants of communicable disease control. We seek to contribute to this understanding by assessing the role of international multilateral organisations in shaping communicable disease burdens across the world. As one of the world’s leading international financial institutions, the International Monetary Fund (IMF) is uniquely positioned to shape the developmental trajectories of fiscally distressed nations through its conditional lending schemes, known as ‘structural adjustment programmes’. In particular, the Fund plays a pivotal role in shaping state capacities to manage pandemics by moulding the institutional infrastructure of local health and welfare systems [1].

Previous research has shown that the IMF-sponsored pursuit of short-term economic goals at the expense of long-term public investments by financially constrained governments can undermine local health systems via fiscal austerity and rapid privatisation reforms. For instance, the curtailing of healthcare coverage and sapping of resources from primary care can weaken the quality of care provision [2, 3]. More generally, IMF-mandated policy reforms—known as ‘conditionalities’—have been associated with reduced state capacities and declining population health [4–6]. The availability of doctors, nurses, community healthcare workers, and associated laboratory and hospital infrastructure are known to be affected by IMF interventions, especially when it comes to tuberculosis and broader communicable disease control [7, 8]. Infectious disease burdens are known to depend on health infrastructures but also on broader social and institutional determinants of health, all of which are impacted by the Fund’s wide-ranging policy reform packages [1]. However, previous studies of the IMF-infectious disease link have typically been confined to specific time periods and geographical areas and have not made use of recent methodological advances in the study of the causal effects of the IMF programmes.

A separate literature has shown that the rapid privatisation of state-owned enterprises can damage population health by increasing unemployment and social insecurity, as well as eroding the public provision of various social goods, including access to healthcare [9, 10]. However, research on the nexus between privatisation and mortality through the lens of structural adjustment remains scarce, and virtually nothing is known about how IMF-mandated privatisation reforms affect disability and death burdens from communicable diseases. Our paper fills this gap by using previously unavailable data and a compound instrumental variable to derive statistically robust effect estimates.

## Methods

Our outcome variable is the country-level age-standardised mortality rate from respiratory infections and tuberculosis (TB) per 100,000 population between 1990 and 2017. Taken together, these causes of death make up category A.2 within the framework of the Global Burden of Diseases, Injuries, and Risk Factors Study, the methodology of which is employed to generate comparable age-standardised mortality metrics across country-years. The case definition of the outcome variable includes all forms of tuberculosis–including latent, drug susceptible, and drug-resistant infections–together with lower and upper respiratory infections as well as otitis media, all of which have been bacteriologically confirmed or clinically diagnosed. The data are compiled and coordinated by the Institute for Health Metrics and Evaluation [11].

We employ two sets of treatment variables to assess the effects of structural adjustment, drawn from the recently established (and continuously updated) IMF Monitor database [12]. On the one hand, we use a dichotomous indicator of whether a country is under an IMF programme to estimate an overall average treatment effect of IMF intervention. On the other hand, to further probe the specific nature of structural loan conditions and their relation to the outcome variables, we assess the role of IMF-mandated privatisations of state-owned enterprises. The latter measure of specific conditionalities has been unavailable to previous studies of the health impacts of IMF programmes.

Our analysis is conducted on a sample of 187 countries. Countries were included in the analysis on the basis of their IMF membership–by virtue of which they are eligible for economic assistance from the Fund–as well as availability of data on the outcome variable over the sample period. However, different countries have different propensities to participate in IMF programmes, depending on a variety of underlying economic and political factors which may also be related to the countries’ health outcomes. For instance, wealthier (and healthier) countries are comparatively less likely to need IMF assistance compared to poorer countries, whilst financial aid is most often sought by low- and middle-income countries that are in the throes of a financial crisis (as measured by contracting GDP). These are also the countries with the highest infectious disease burdens. However, the IMF is unlikely to undertake major interventions in politically unstable countries (such as those facing civil wars). Moreover, the health impacts of IMF programmes can also depend on certain compositional features of the client country’s population, such as levels of education, which may mediate or modify upstream policy reforms. For instance, highly educated parents may be better positioned to protect their children against the harms of a contracting healthcare system.

Against this backdrop, our economic control variables are gross domestic product (GDP) per capita, measured in constant 2010 US dollars [13], a binary financial crisis indicator [14], and foreign reserves in months of imports [13]. Our political control variables include a general democracy index [15] and a more refined measure of egalitarian democracy [16], a coup d’état indicator [17] as a measure of political instability, and United Nations General Assembly (UNGA) voting alignment with the G7 countries [18]. The latter variable is construed as a proxy for geo-strategic alignment and is known to be predictive of IMF programme participation and potentially of the types of conditionalities received by borrowing countries [19]. We also control for average years of completed education in the female population aged 25–29 [11]. Finally, to test the hypothesis according to which much of the proposed impact propagates through health systems, we control for the number of hospital beds per 1,000 population [13]. Descriptive statistics are shown in Table 1. As is evident from Table 1, there are several missing values in our data, notably when it comes to the control variables. The missingness patterns are such that multiple imputation of missing data is not viable. Since our instrumental variable is meant to be robust to unmeasured confounding, our baseline model is therefore run without additional controls to avoid losing too many observations at once. The robustness checks involve adding and removing control variables one at a time to assess corresponding changes in the estimated treatment effect.

**Table 1:** Descriptive statistics.

Statistic	N	Mean	St. Dev.	Min	Max
Communicable disease mortality rate	5,460	96	103	5	613
IMF programme	5,165	0.3	0.5	0.0	1.0
IMF-mandated privatisation	5,131	0.1	0.5	0.0	12
GDP per capita	4,733	11,561	17,103	164	111,968
Financial crisis	5,236	0.1	0.2	0.0	1.0
Foreign reserves	3,932	4.4	4.7	0.002	79
Democracy	4,909	3.5	6.6	−10	10
Egalitarian democracy	4,097	0.4	0.2	0.03	0.9
Coup d’état	4,396	0.02	0.1	0.0	1.0
UNGA voting alignment	5,012	−1.6	1.0	−3.9	1.4
Mean years of female education	5,404	8.9	4.0	0.5	16
Hospital beds per 1,000 population	2,694	4.1	3.1	0.01	20

Notes: The mortality rate from communicable diseases is age-standardised per 100,000 population and refers to deaths from respiratory infections and tuberculosis. The second column lists the number of observed country-years. The privatisation variable counts the total number of privatisation conditionalities imposed on a borrowing country by the IMF. The general democracy index ranges from −10 to 10. The egalitarian democracy index ranges from 0 to 1.

We posit the following data-generating process:

$$Y_{it}=T_{i,t-k}\beta +X_{it}\theta +\mu_{i}+\phi_{t}+\varepsilon_{it},$$ (1)

where *Y*_*it*_ denotes one of the two alternative outcome variables as measured in country *i* at time *t*; *T*_*i*,*t*−*k*_ is one of our two dichotomous treatment variables indicating whether a country participates in an IMF programme or whether it has implemented an IMF-mandated privatisation reform, lagged by *k* ∈ {1,5,10} years to allow for dynamic effects to manifest; *X*_*it*_ is a vector of control variables; *μ*_*i*_ captures time-invariant country-specific effects; *ϕ*_*t*_ measures time-fixed effects; and *ε*_*it*_ is a stochastic error term. Our principal quantity of interest is *β*, which is a causal effect parameter to be estimated: it measures the number of additional respiratory disease deaths per 100,000 population associated with the counterfactual contrast between the IMF intervening in a country versus the IMF not intervening. Below, we refer to this as the number of ‘excess deaths’ per 100,000 population. A common approach to estimating *β* is through the use of two-way fixed effects models that isolate variation within units over time whilst controlling for aggregate time trends. This has the virtue of eliminating all bias induced by time-invariant confounders. However, some countries might be more likely to seek the IMF’s assistance than other countries, and this might generate a spurious association between structural adjustment programmes and our outcomes of interest that cannot be addressed through the sole use of a fixed effects model.

The possibility of such a spurious association can be more intuitively illustrated as follows. Consider two doctors, A and B. Doctor A is a highly skilled clinician who, on the basis of a strong record, is only assigned patients whose treatment other doctors find too difficult or challenging and whose survival chances are low. Doctor B, on the other hand, is less competent and is therefore only assigned easier cases. If ‘success’ is defined in terms of patient survival, a naive comparison of the success rates of doctors A and B can lead to the faulty conclusion that doctor B is more competent than doctor A since the former’s patients have a higher marginal chance of survival. Such a conclusion fails to take into account the severity of the disease that each doctor is treating: since doctor A only treats the sickest patients, his or her success rate is bound to be low compared to doctor B, who only works with easily treated patients. In a similar vein, the IMF can be viewed as a doctor who intervenes across the world to treat (financially) ailing patients (countries), but caution is warranted in interpreting subsequent health outcomes without controlling for other factors that make some units more likely to receive the treatment than others (that is, pre-treatment ‘disease severity’). Previous research has indeed shown that failing to account for such bias in the study of IMF programmes almost invariably leads to meaningless results [20–22].

One way to address this concern is to adjust for as many confounders (*X*) as possible in our model. However, in an observational study of such a complex matter, it is hard to know whether all sources of confounding are adequately addressed. A different and more compelling solution is to construct an instrumental variable, *Z*, which is correlated with the treatment, *T*, but uncorrelated with any other variables in the causal system, thereby isolating quasi-random variation in *T*. For an intuitive understanding of this concept, consider the following example. Suppose we are interested in the causal effect of alcohol consumption on liver disease. We could simply compute the association between these two variables in a population but we are likely to be concerned that the corresponding effect estimate is biased, especially if there is a third unobserved factor that influences both alcohol consumption and liver disease. Such a factor might include an underlying propensity to health destructive behaviour or broader social determinants of health that drive such behaviours. To avoid this problem, we might exploit (e.g., regional) variation in the price of alcohol as an instrumental variable: the price of alcoholic beverages will affect drinking behaviour—but it is unlikely to affect liver disease other than through its effect on drinking behaviour. In other words, it is not correlated with other confounding factors and thus isolates what is known as ‘exogenous’ variation in the independent variable.

**Fig 1:**
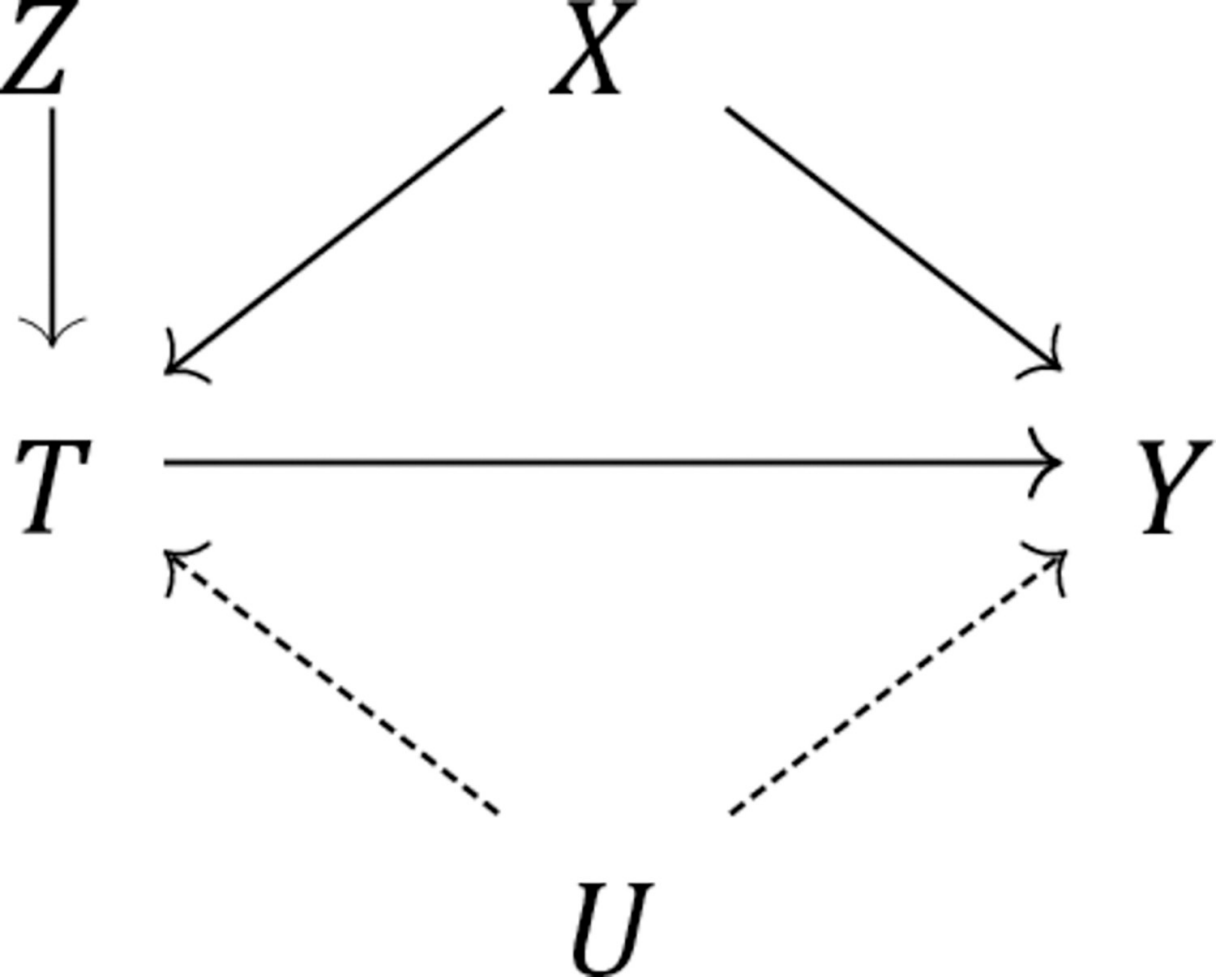
Causal graph depicting the effect of the treatment variable (*T*) on the outcome (*Y*), identified via a compound instrument (*Z*), net of both measured covariates (*X*) and unmeasured confounders (*U*).

In our case, as per Fig 1, an instrumental variable (*Z*) is a variable that is predictive of the intervention of interest (*T* = IMF programmes) but that is uncorrelated with unmeasured confounders (*U*). These two criteria are known as the ‘relevance criterion’ (the instrument needs to be relevant to, i.e., have an impact on, the treatment) and the ‘exclusion criterion’ (the instrument must not be associated with other variables in the causal system under consideration), respectively. To obtain such an instrument, we follow recent methodological advances in the study of structural adjustment [20, 21] by adopting an instrument derived from the Fund’s annual budget constraint, as measured by the number of countries with an IMF programme in a given year [22]. The IMF’s annual budget constraint should meet the two key criteria for an instrumental variable: it is clearly related to IMF programmes, but there is no reason to suspect that it is correlated with country-specific health profiles. In other words, whether or not the IMF is experiencing liquidity problems is not a function of the state of health in any given client country (just as the price of alcohol should not depend on any given individual’s propensity to drink). Moreover, previous research has shown that the IMF is more likely to impose harsher loan conditions when it faces liquidity concerns, regardless of the client in question [20–22]. This allows us to specifically investigate ‘high-dose’ interventions by the Fund. In more technical terms, our identification strategy relies on a compound instrument derived from the interaction between the country-specific average exposure to IMF programmes over the sample period and the Fund’s annual budget constraint. This instrument has been carefully evaluated in the extant literature [20, 21]. Further technical methodological details are provided in the S1 File.

Finally, we include two-way fixed effects in our instrumented panel regression model. As noted above, this has the virtue of eliminating two additional potential sources of bias: (1) time-invariant confounders, such as long-run institutional factors that might affect a country’s propensity to participate in IMF programmes whilst also being correlated with the country’s health performance, such as the organisation of health systems or political institutions, and (2) time-varying aggregate trends that affect all countries simultaneously. Over the study period, significant resources have invested, nationally and internationally, for control of tuberculosis and other infectious diseases. This has yielded downward-sloping trends in a variety of infectious disease outcomes at the aggregate level. The inclusion of time-fixed effects ensures that our model is not merely picking up variation pertaining to such aggregate trends, but rather that it isolated exogenous variation pertaining to IMF interventions.

Given that we cannot empirically verify whether or not the instrument is strictly exogenous, the persistence of unmeasured residual confounding is possible. To address this concern, we also conduct a simple yet comprehensive non-parametric sensitivity analysis that allows us to quantify the amount of unmeasured confounding that would in theory be required to eliminate our estimated treatment effect *β*^ˆ^. As per Fig 1, let *U* denote an unmeasured confounder. Then the bias factor, B, is defined as the difference between *β*^ˆ^ and what *β*^ˆ^ would have been had we controlled for *U* as well. Assuming *U* is binary, we define

γ=E(Y|U=1,T)−E(Y|U=0,T)

as the net effect of the unmeasured confounder on the outcome and

δ=P(U=1|T=1)−P(U=1|T=0)

as the difference in the prevalence of the unmeasured confounder between the treatment and control groups. Then the bias factor is the product of these two sensitivity parameters: B = *γ*·*δ* [23, 24]. In assessing the sensitivity of our model coefficients to unmeasured confounding, we ask how large *γ* would have to be in order to reduce our estimated effect size *β*^ˆ^ to zero. We address this question by visualising how B changes as the two sensitivity parameters (co-)vary across a range of possible values.

## Results

**Fig 2:**
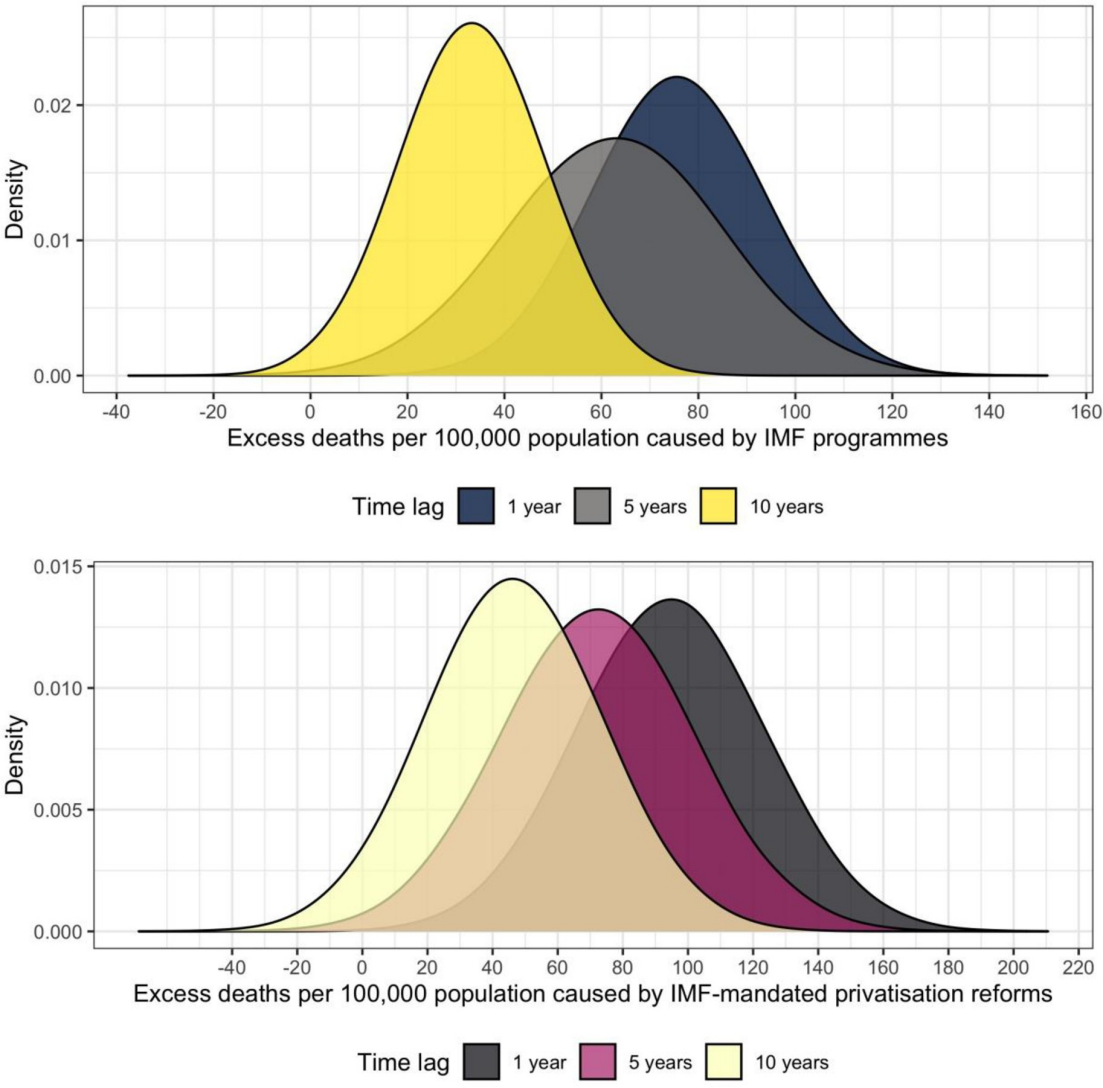
The figure visualises the estimated excess mortality burden from respiratory infections and tuberculosis per 100,000 population caused by IMF programmes as a whole (top) and IMF-mandated privatisation reforms (bottom). The estimates are derived from two-way fixed effects instrumental variable regression models in which within-country changes in mortality rates across units with and without IMF programmes or privatisation conditionalities are calculated. First differences in the outcome variable are then used to estimate excess death rates.

Fig 2 displays our baseline results. As seen in the top half of the figure, we find that IMF programmes as a whole exert a substantively large impact both on mortality rates from respiratory infections and TB per 100,000 population, notably in the short (*β*^ˆ^ = 76 excess deaths, 95% CI: 42–110) and medium (*β*^ˆ^ = 63 excess deaths, 95% CI: 20–106) term. In the long run, the effect dissipates somewhat and is harder to identify precisely, as evidenced by smaller effect sizes and widening confidence intervals (*β*^ˆ^ = 33 excess deaths, 95% CI: 4–62). As seen in the bottom half of the figure, this aggregate effect appears to be driven by IMF-mandated privatisation reforms, which generate large excess death burdens from communicable diseases (*β*^ˆ^ = 95 excess deaths after 1 year, 95% CI: 39–151). Also in this case, we find that the effect weakens after a decade (*β*^ˆ^ = 46 excess deaths after 10 years, 95% CI: -6–98).

To assess any confounding from observed variables, we check the robustness of our parameter to additional control variables. To avoid multicollinearity and the loss of too many observations at once due to missing data, we add and remove these controls one by one and inspect the corresponding change in the treatment coefficient. To avoid unnecessary clutter, we limit our sensitivity analysis to the short-term effects identified above and omit all but key quantities of substantive interest (i.e., the estimated treatment effects). As displayed in Table 2, we find that structural adjustment remains a robust predictor of our outcome variable. The greatest attenuation in the estimated baseline treatment effects occurs when controlling for foreign reserves—which is an important predictor of selection into IMF programmes [22]. As shown in the last row of the table, we also find evidence that health systems are an important mechanism through which these adjustments impact health, with estimates being attenuated by nearly one half when controlling for the number of hospital beds per 1,000 population. This is not the case for privatisation reforms, the coefficient for which remains stable despite increasing estimation uncertainty. We note, however, that the hospital beds per capita models are based on a substantially reduced sample size (total *N* = 2,517) due to missing data and should therefore be interpreted with some caution.

**Table 2:** Instrumented two-way fixed effects control models.

Control variable	IMF_*t*−1_ coefficient	Privatisation_*t*−1_ coefficient
Log of GDP per capita	63	80
	(24, 102)	(8, 152)
Financial crisis	77	95
	(42, 112)	(40, 150)
Foreign reserves	47	86
	(18, 76)	(17, 155)
Democracy	68	87
	(35, 101)	(30, 144)
Egalitarian democracy	87	87
	(30, 108)	(18, 156)
Coup d’état	75	76
	(30, 120)	(9, 143)
UNGA voting alignment	73	89
	(40, 106)	(32, 146)
Female education	79	97
	(46, 112)	(42, 152)
Hospital beds per 1,000 population	31	93
	(13, 49)	(−1, 187)

Notes: The outcome variable is the age-standardised mortality rate from respiratory infections and tuberculosis per 100,000 population. Each row is a separate two-way fixed-effects regression wherein the effect of IMF programmes or IMF-mandated privatisation reforms on the outcome is adjusted for the control variable listed in the first column. All models are also adjusted for country- and time-fixed effects. The two treatment variables, lagged by one year, are instrumented as described in the Methods section. 95% confidence intervals derived from standard errors consistent with heteroskedasticity, serial correlation, and unit clustering are shown in parentheses below each parameter estimate.

**Fig 3:**
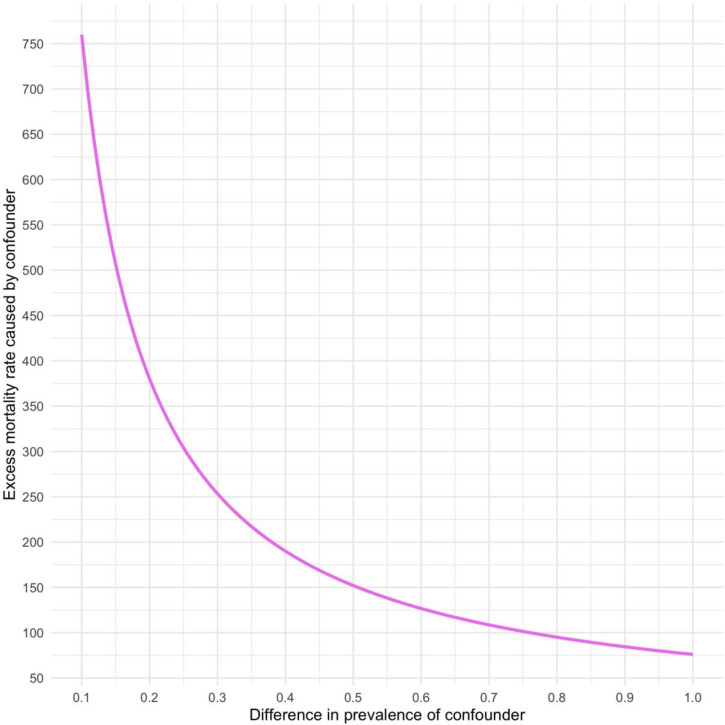
Sensitivity analysis plot to assess residual confounding of the estimated effect of IMF programmes on mortality rates from respiratory infections and tuberculosis as per the top half of Fig 2. Values on the solid lines would completely eliminate the estimated effect of IMF programmes. Values above the plotted curves would reverse the sign of the estimated effects.

**Fig 4:**
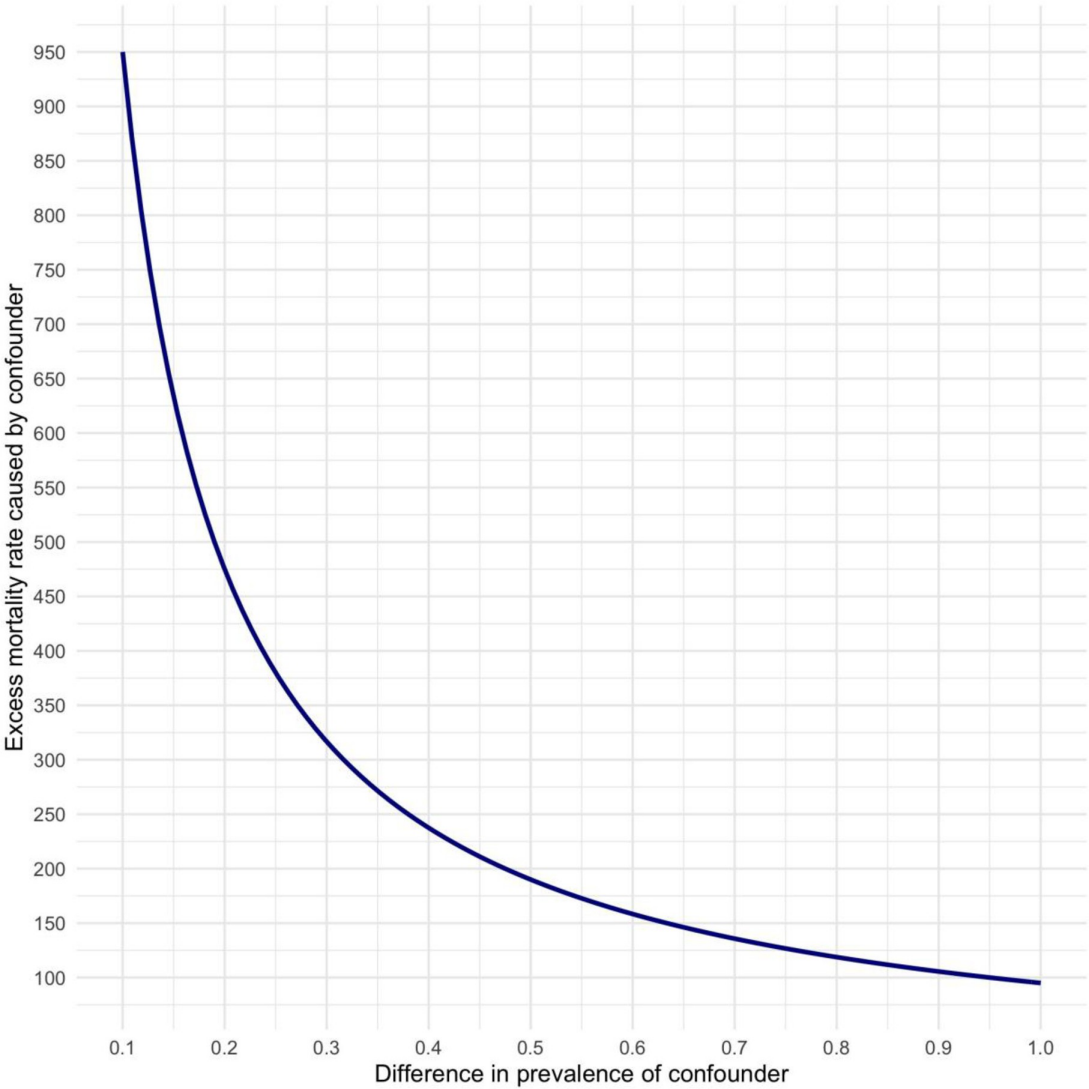
Sensitivity analysis plot to assess residual confounding of the estimated effect of IMF-mandated privatisation reforms on mortality rates from from respiratory infections and tuberculosis as per the bottom half of Fig 2. Values on the solid lines would completely eliminate the estimated effect of privatisation reforms. Values above the plotted curves would reverse the sign of the estimated effects.

Given the observational nature of our study, the persistence of unmeasured residual confounding is possible. To address this concern, we conduct a simple non-parametric sensitivity analysis that allows us to quantify the amount of unmeasured confounding that would in theory be required to eliminate our estimated short-run causal effects, as described above. The results of this analysis are visualised in Figs 3 and 4. The Y-axis quantifies the net effect of *U* on the outcome variable that would be required to completely eliminate the estimated causal effect of structural adjustment programmes. In light of our instrumented treatment variable, we believe it is plausible that the amount of residual confounding remains small and that the difference in prevalence of any confounder between treatment and control groups should be minimal. As such, the most likely magnitude of *δ* would be at the lower end of the X-axis in Fig 3. If *δ* = 0.1, *U* would have to cause over 750 excess communicable disease deaths per 100,000 population to nullify the effect of IMF programmes. Even higher net effects, exceeding 900 excess deaths per 100,000, would be required to nullify the impact of privatisation conditionalities, as seen in Fig 4. Also at higher values of *δ*, an inordinate amount of unmeasured confounding would be needed to cast serious doubt on our effect estimates.

## Concluding discussion

Our analysis provides novel evidence from previously unavailable cross-national panel data, linking the IMF’s policy interventions to poor health outcomes. We corroborate earlier studies and hypotheses surrounding this topic, yet we offer new empirical insights. Our main finding is that IMF programmes, and IMF-mandated privatisation reforms in particular, increase avoidable burdens of illness and death from respiratory infections and TB. Although we are unable to specify the specific mechanisms by which the estimated causal effects take place, we find that a substantial portion of the aggregate effect seems to be mediated by how IMF programmes impact local health systems, as measured by the number of available hospital beds per 1,000 population. This is in line with the extant literature, which suggests that IMF-mandated reductions in health and public sector spending affect the quality of primary care in addition to exerting durable influence on the social determinants of health [1]. For lack of data, we are unable to probe the mechanisms any further, though we note that the rapid privatisation of state-owned enterprises has previously been linked to turbulent labour market conditions, high levels of social insecurity and stress, and weaker public institutions [9, 10]. Such insights lend credence to our findings.

We note that the observational nature of our analysis precludes any guarantee of strictly unbiased model estimates. However, the sensitivity analysis suggests that an unusual amount of unmeasured confounding would be required to cast serious doubt on our substantive findings. We thus consider our results as providing an important empirical basis for future policy-making and research. We therefore conclude that structural adjustment programmes, as currently designed and implemented, are harmful to population health by increasing global communicable disease burdens and must be rethought and reformed.

## Supporting information

S1 File (DOCX)

## References

[1] Kentikelenis AE. Structural adjustment and health: a conceptual framework and evidence on pathways. Soc Sci Med 2017; 187: 296–305. doi: 10.1016/j.socscimed.2017.02.021 28238540

[2] Kentikelenis AE, Stubbs TH, King LP. Structural adjustment and public spending on health: Evidence from IMF programs in low-income countries. Soc Sci Med 2017; 126: 169–76. 10.1016/j.socscimed.2014.12.027 25576997

[3] Stubbs TH, Kentikelenis AE, Stuckler D, McKee M, King LP. The impact of IMF conditionality on government health expenditure: A cross-national analysis of 16 West African nations. Soc Sci Med 2015; 174: 220–227. 10.1016/j.socscimed.2016.12.016 28087084

[4] Forster T, Kentikelenis AE, Stubbs TH, King LP. Globalization and health equity: The impact of structural adjustment programs on developing countries. Soc Sci Med 2019; online first: 112496. doi: 10.1016/j.socscimed.2019.112496 31515082

[5] Reinsberg B, Kentikelenis AE, Stubbs TH, King LP. The world system and the hollowing out of state capacity: How structural adjustment programs affect bureaucratic quality in developing countries. Am J Sociol 2019; 124(4): 1222–1257.

[6] Stuckler D, Basu S. The International Monetary Fund’s effects on global health: before and after the 2008 financial crisis. Int J Health Serv 2009; 39(4): 771–781. doi: 10.2190/HS.39.4.j 19927414

[7] Kentikelenis AE, Karanikolos M, Reeves A, McKee M, Stuckler D. Greece’s health crisis: from austerity to denialism. Lancet 2009; 383(9918): 748–753. 10.1016/S0140-6736(13)62291-6 24560058

[8] Stuckler D, King LP, Basu S. International Monetary Fund programs and tuberculosis outcomes in post-communist countries. PLoS Med 2008; 5(7): e143. doi: 10.1371/journal.pmed.0050143 18651786 PMC2488179

[9] Azarova A, Irdam D, Gugushvili A, Fazekas M, Scheiring G, Horvat P, et al. The effect of rapid privatisation on mortality in mono-industrial towns in post-Soviet Russia: a retrospective cohort study. Lancet Public Health 2017; 2(5): e231–e238. doi: 10.1016/S2468-2667(17)30072-5 28626827 PMC5459934

[10] Scheiring G et al. The gendered effects of foreign investment and prolonged state ownership on mortality in Hungary: an indirect demographic, retrospective cohort study. *Lancet Global Health* 2018; 6(1): e95–e102. doi: 10.1016/S2214-109X(17)30391-1 29191434

[11] Global Burden of Disease Collaborative Network. Global Burden of Disease Study 2017: Socio-demographic Index 1970–2017. Seattle, United States: Institute for Health Metrics and Evaluation, 2018.

[12] Kentikelenis AE, Stubbs TH, King LP. IMF conditionality and development policy space, 1985–2014. Rev Int Political Econ 2016; 23(4): 543–582.

[13] World Bank. World Development Indicators. http://data.worldbank.org/data-catalog/world-development-indicators. Accessed 1 June 2021.

[14] Laeven L, Valencia F. Systemic Banking Crises Database. IMF Econ Rev 2013; 61: 225–270.

[15] Center for Systemic Peace. The Polity Project. https://www.systemicpeace.org/polityproject.html. Accessed 1 June 2021.

[16] Quality of Government Database. https://www.gu.se/en/quality-government/qog-data. Accessed 1 June 2021.

[17] Powell JM, Thyne CL. Global Instances of Coups from 1950 to 2010: A New Dataset. J Peace Res 2011; 48(2): 249–259.

[18] Bailey M, Strezhnev A, Voeten E. Estimating Dynamic State Preferences from United Nations Voting Data. J Conflict Resolut 2015; 61(2): 430–456.

[19] Sturm JEA, Vreeland JR. Politics and IMF Conditionality. J Conflict Resolut 2015; 59(1): 120–148.

[20] Lang V. The Democratic Deficit and Its Consequences: The Causal Effect of IMFPrograms on Income Inequality. *Rev Int Organ* 2020; 10.1007/s11558-020-09405-x.

[21] Stubbs TH, Reinsberg B, Kentikelenis AE, King LP. How to evaluate the effects of IMF conditionality: An extension of quantitative approaches and an empirical application to public education spending. Review Int Organ 2020; 15(1): 29–73.

[22] Vreeland JR. The IMF and Economic Development. Cambridge: Cambridge University Press; 2003.

[23] VanderWeele TJ, Arah OA. Bias formulas for sensitivity analysis of unmeasured confounding for general outcomes, treatments, and confounders. Epidemiol 2011; 22(1): 42–52. 10.1097/EDE.0b013e3181f74493 PMC3073860 21052008

[24] VanderWeele TJ. Explanation in Causal Inference: Methods for Mediation and Interaction. New York: Oxford University Press; 2015.

