# Supplementary material for: Structural adjustment programmes and infectious disease mortality
Source: PLoS One. 2022 Jul 15;17(7):e0270344. doi: 10.1371/journal.pone.0270344 (PMC9286264; doi:10.1371/journal.pone.0270344)
Supplement: S1 File — (DOCX) [file pone.0270344.s001.docx]

# **Supplementary information**

Technically, our identification strategy relies on a compound instrument derived from the interaction between the country-specific average exposure to IMF programmes over the sample period and the Fund’s annual budget constraint, approximated by the number of countries with an IMF programme in a given year [22]. This instrument meets the relevance criterion insofar as the IMF is likely to impose more stringent loan conditions when facing liquidity concerns. Previous research has indeed shown that the IMF’s budgetary constraint is predictive of harsher loan conditions, regardless of the client country [20, 22]. The proposed instrument also meets the exclusion criterion insofar as the Fund’s aggregate annual budget constraints are independent of any given country, such that unit-specific shocks that deviate from a country’s long-run average exposure to structural adjustment result from a treatment assignment mechanism that is orthogonal to (i.e., uncorrelated with) any given country’s potential (or counterfactual) outcomes. In other words, the outcome of interest in countries with varying propensities to participate in IMF programmes will not be affected by changes in the Fund’s budgetary constraint other than through the impact of structural adjustment. Our identification strategy is visually summarised in Figure 1, where the conditional independence assumption obtains by deploying *Z* as a source of exogenous variation in *T* that helps us identify a local average treatment effect. Thus, the joint usage of an instrumental variable and of country- and time-fixed effects provides a rigorous framework for causal identification.

We thus obtain a two-stage regression model with the following selection equation:


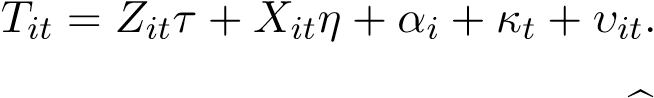
 (2)

We then re-specify the model in equation (1) as follows, with *T* being a vector of fitted values from equation (2):


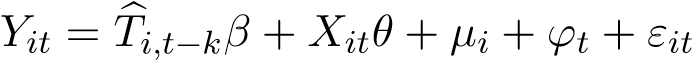
*.* (3)

To empirically assess the strength of the chosen instrument, we compare the model in equation (2) to a restricted first-stage regression in which the effect *τ* of *Z* on *T* is set to be null, obtaining a *χ*^2^ test statistic of 53.641 on 1 degree of freedom (*p <* 0*.*001). Our alternative instrument targeting privatisation conditionalities also passes the required significance threshold, with a *χ*^2^ test statistic of 40.464 on 1 degree of freedom (*p <* 0*.*001). Hence, in both cases, *Z* comfortably satisfies convention criteria for strong instruments, meaning they do predict the treatment. We control for the endogenous relation between *T* and *Y* potentially induced by any time-invariant propensity of countries with a prior health disadvantage to select into IMF programmes by adjusting for country-fixed effects, whereas year-fixed effects help account for broader time trends that affect all countries simultaneously. All variance estimators are robust with respect to serial autocorrelation, heteroskedasticity, and country-level clustering effects. All analyses are conducted in R, version 4.0.2.
